# Perceptual Color Characterization of Cameras

**DOI:** 10.3390/s141223205

**Published:** 2014-12-05

**Authors:** Javier Vazquez-Corral, David Connah, Marcelo Bertalmío

**Affiliations:** 1 Information and Communications Technologies Department, Universitat Pompeu Fabra, Roc Boronat 138, Barcelona 08018, Spain; E-Mail: marcelo.bertalmio@upf.edu; 2 Centre for Visual Computing, University of Bradford, Bradford BD7 1DP, UK; E-Mail: d.connah@bradford.ac.uk

**Keywords:** color characterization, perceptual correction, camera sensor response

## Abstract

Color camera characterization, mapping outputs from the camera sensors to an independent color space, such as *XY Z*, is an important step in the camera processing pipeline. Until now, this procedure has been primarily solved by using a 3 × 3 matrix obtained via a least-squares optimization. In this paper, we propose to use the spherical sampling method, recently published by Finlayson *et al.*, to perform a perceptual color characterization. In particular, we search for the 3 × 3 matrix that minimizes three different perceptual errors, one pixel based and two spatially based. For the pixel-based case, we minimize the CIE Δ*E* error, while for the spatial-based case, we minimize both the S-CIELAB error and the CID error measure. Our results demonstrate an improvement of approximately 3% for the Δ*E* error, 7% for the S-CIELAB error and 13% for the CID error measures.

## 1. Introduction

**Figure 1.**
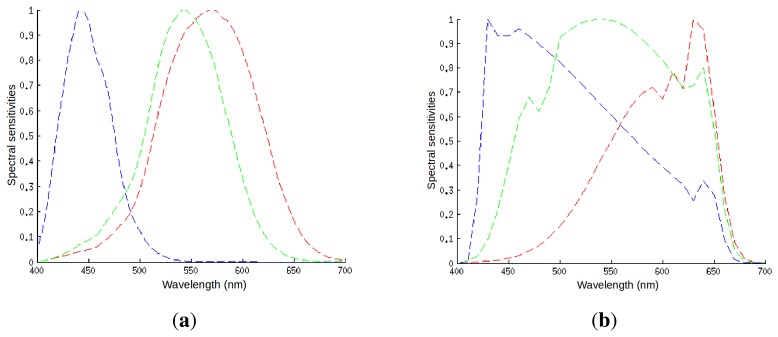
Spectral sensitivities of: (**a**) the three types of cones in a human eye; and (**b**) a typical digital camera.

At first glance, it would seem that for a camera to accurately capture colors matching our perception, the color triplets obtained by the camera sensor(s) should correspond to the cone responses of the human visual system. However, this is never the case, as Figure 1 shows, and since the spectral sensitivities of sensor and cones are different, the responses must also be different.

There are several reasons for this difference, like the fact that it is difficult to tune the spectral response of the pigments or dyes of the color filter arrays and that having spectral sensitivities with a large amount of overlap (as in the responses of medium and long wavelength cones) would not be practical from a signal-to-noise point of view [1]. However, while emulating cone responses is not practical for image capture, it is essential in the subsequent processing of the image signal: the stimulus the scene would have produced in the human visual system must be estimated as accurately as possible [1]. This is why we must be able to transform the (*R*, *G*, *B*) values of the sensor into (*X*, *Y*, *Z*) tristimulus values, *i.e.*, go from *RGB* into CIE *XYZ*, which we recall is a color space that uses the color-matching functions *x̄*, *ȳ*, *z̄* of a standard observer, obtained from perceptual color-matching experiments.

We can transform (*R*, *G*, *B*) into (*X*, *Y*, *Z*) by imposing the Luther–Ives condition [2]: that the sensor response curves are a linear combination of the color matching functions. Manufacturing processes and the properties of the materials used make it difficult to adjust at will the sensor response curves, and the Luther–Ives condition is usually not met in practice [3]. Despite this fact, a three-channel camera with three arbitrary sensor response curves is able to estimate the tristimulus values of an object as long as the object's spectral reflections are always composed of three principal components and they do not change steeply with respect to wavelength [3]. This implies that with a linear transformation, we can go from the observed (*R*, *G*, *B*) triplet to its corresponding (*X*, *Y*, *Z*) tristimulus value, and this process is called color characterization. We must point out that the transform itself could also be a non-linear mapping [4,5].

While the non-linear approach generally uses more parameters and, hence, has greater potential to reduce error, the linear approach remains very popular. This is due to two key advantages: (1) with linear methods scaling the *RGB* responses results in a direct scaling of *XYZ* values; and (2) linear methods preserve the hue-planes of the input *RGB* image [6].

A standard way for camera manufacturers to perform color characterization is the following [3]:

(1) Build a set of *n* test patches of representative or important colors.
(2) Under controlled conditions, with a known illuminant (e.g., D65), measure the tristimulus values of the patches with a tristimulus colorimeter, obtaining (*X_i_*, *Y_i_*, *Z_i_*), 1 ≤ *i ≤ n*.
(3) Under the same conditions, use the camera to measure the (*R*, *G*, *B*) values of the patches, obtaining (*R_i_*, *G_i_*, *B_i_*), 1 ≤ *i* ≤ *n*.
(4) A linear transformation, a 3 × 3 matrix called the colorimetric matrix, gives an estimated tristimulus value (*X̂_i_*, *Ŷ_i_*, *Ẑ_i_*) from (*R_i_*, *G_i_*, *B_i_*). This matrix is computed so as to minimize the total visual color difference *J*, which is a weighted sum of the color differences Δ*E* (computed, for instance, in a CIE perceptual uniform color space) between the target tristimulus (*X_i_*, *Y_i_*, *Z_i_*) and its estimate (*X̂_i_*, * Ŷ_i_*, *Ẑ_i_*), for each patch *i*, 1 ≤ *i* ≤ *n*: $J={\text{∑}}_{i=1}^{n}w_{i}\Delta E\left(X_{i},Y_{i},Z_{i},{\hat{X}}_{i},{\hat{Y}}_{i},{\hat{Z}}_{i}\right)$, where *w_i_* are the weights for the different patches. The colorimetric matrix is usually obtained through least-squares minimization.

In [7], it is noted that the above procedure has the problem that the white point is not preserved, *i.e.*, white in *RGB* is not mapped to white in the CIE *XYZ* color space, where white in *XYZ* represents the *XYZ* response to a perfect reflecting diffuser under a specified illuminant; an additional term can be added to J in order to prevent this [8], and more accurate and robust techniques have also been proposed [9].

Some cameras come with several pre-set matrices computed under different illuminations. For instance, using the matrix for fluorescent lighting removes a noticeable green cast that would otherwise be present if we used a matrix computed with a standard illuminant, like D65 or D50; other pre-sets may correspond, for instance, to a “film look” (with de-saturated colors) or may give a very vivid color palette. These pre-set matrices can also be adjusted manually, so as to achieve a certain image look, since changing the colorimetric matrix affects hue and saturation (the white point is preserved, though, and color matrix adjustment must not be confused with white balance). Video cameras were the first to incorporate the possibility of modifying the colorimetric matrix, so that multiple cameras in live broadcasts could be color matched and there appeared no color jumps when switching from one camera to another [10].

In this paper, we propose an approach to characterization, which retains the advantages of linear transforms, while explicitly minimizing perceived differences in perceptual color spaces. The approach works in two stages: in the first stage, an *RGB* to *XYZ* transform is computed that minimizes the least-squares error in the usual way; in the second stage, this transform is adjusted to reduce a defined perceptual measure. This adjustment is done using the method of spherical sampling of Finlayson *et al.* [11], which generates a set of discrete putative transforms that deviate systematically from the first transform. We will show that choosing the best of these transforms to minimize the perceptual error results in an improved characterization performance. Let us stress here that, to the authors' knowledge, this is the first time spherical sampling has been used out of the spectral sharpening domain. This may open the door to the application of spherical sampling to further applications in different domains.

This is valid for any kind of digital camera, be it for video or still photography, using CCD or CMOS sensors. Our method provides camera manufacturers and also color researchers and professional photographers and cinematographers with a simple way to record images, whose colors are more faithful, in a perceptual sense, to those in the real world scene where the images were taken.

## 2. Background

### 2.1. Sources of Error

The error in the characterization, *i.e.*, the difference between the target and estimated *XY Z* values, is dependent on the sensor sensitivity of the camera, the reflectance spectra of the objects in the scene and the illuminant of the scene. In the case that the *RGB* sensor sensitivities are within an exact 3 × 3 transform of the *XY Z* color matching functions (CMFs) or, equivalently, the *RGB* bases and *XY Z* CMFs span the same linear subspace, then the error in the characterization is guaranteed to be zero [12]. In practice, this condition is rarely met: as we mentioned, there are inherent variations in the sensor manufacturing process, as well as conflicting design considerations, such as robustness to noise.

One approach to solving this problem would be to map *RGB* sensors directly to *XY Z* CMFs using the linear transform *T* minimizing:

(1) $$\arg\underset{T}{\min}{\left\|S_{\text{xyz}}-S_{\text{rgb}}T\right\|}_{2},$$

where *S_xyz_* denotes an *m* × 3 matrix of *XY Z* color matching functions sampled at *m* discrete wavelengths, *S_rgb_* is an *m* × 3 matrix of camera *RGB* responses and *T* is a 3 × 3 linear transform. This can be shown to be the best approach when no other information about spectra is known (the maximum ignorance assumption) [13]; *i.e.*, when spectra are pure noise stimuli. However, this is not the case for real spectra, which are both positive and smoothly varying functions of wavelength [14,15]. As such, the structure of reflectances becomes an important factor in defining the optimal transform. For this reason, the minimization is usually performed as:

(2) $$\arg\underset{T}{\min}{\left\|M_{\text{xyz}}-M_{\text{rgb}}T\right\|}_{2},$$

where *M_xyz_* denotes an *n* × 3 matrix representing *XY Z* values for a set of illuminants and reflectances and *M_rgb_* is an *n* × 3 matrix representing the *RGB* values for the same set of illuminants and reflectances.

In a world where reflectances can be described by three, or fewer, parameters in a linear model, the characterization process is again guaranteed to result in zero error [16]. In practice, this condition is not met: real reflectances are described by more than three parameters [17], and the characterization error will be non-zero.

In addition to these fundamental sources of error, the imaging process will contain various sources of noise. These factors are not considered in our coverage here.

### 2.2. Perceptual Error

The goal of characterization is to ensure that colors remain stable regardless of the imaging device used. Ultimately, when considered with a display device as part of the color management pipeline, the captured colors should appear to be as similar to the original scene as possible. The implication for the characterization error is that errors should reflect the perceived difference between colors.

The light entering the human eye is firstly captured by the photoreceptors and is immediately subject to neural processing; this comes in the form of adaptation, spatial channel mixing and both pooling and contrasting responses across receptors, even before the visual signal has left the retina. Interactions between neurons in the visual cortex and at higher processing sites mean that relationships between perceived colors are complex, and the color of a stimulus cannot be defined without taking into account its spatial surrounds and context.

Some of the behavior of the human visual system can be predicted by “color appearance models”. These perceptual models approximate the transformation from raw cone-responses (which can be derived from *XY Z* values) to perceptual attributes, such as lightness, colorfulness and hue. An early color appearance model, the CIELAB color space [18], was derived specifically to map colors into a space where color differences correlate closely with Euclidean distances measured in CIELAB. Other more recent models, such as CIECAM02 [19], provide predictors of the perceptual attributes of the colors, but CIELAB color difference formulae (which measure Euclidean distance in CIELAB space) are still used extensively for color difference calculations, and are being incrementally improved alongside the color appearance models, e.g., CIE Δ*E*_00_ [20].

Color appearance models may take into account the non-linear response of the visual system to light transduced by the photoreceptors, including effects, such as adaptation and opponent color mechanisms, but they have a relatively basic notion of spatial relationships between colors. To model these effects, multiple image-difference predictors and image-appearance models have been proposed. These include the relatively simple S-CIELAB model, which incorporates spatial filtering into the CIELAB calculation [21], up to more complex models, such as iCAM [22], which includes spatial filtering and local adaptation in a more complete color appearance model.

Recently, some perceptually-based image quality metrics have been presented. In particular, the Color Image Difference (CID) quality measure [23] computes a color difference metric for a complete image, taking into account multiple imaging factors in a perceptually-based color space, in particular hue, chroma, lightness, lightness-contrast and lightness-structure.

### 2.3. Linear vs. Non-Linear

The characterization process involves a mapping from one three-vector, *RGB*, to a second three-vector, *XY Z*. This can be done through either linear or non-linear mapping. Non-linear approaches, such as polynomials [4], neural networks [5] or geometric approaches [24], have been favored in some applications, as they introduce a greater number of parameters and, hence, can reduce characterization error. Nonetheless, linear approaches are still popular and are widely employed in default conversions used by color camera manufacturers. Linear transforms, while potentially less accurate, preserve two important properties: scalability and the preservation of hue planes, which we will briefly describe now.

The scalability property means that the characterization is equally valid when the exposure duration of the camera is changed; this is not true for most non-linear characterizations, where a change in exposure duration will scale *RGB* values, but may result in angular shifts in the *XY Z* vectors, which, in turn, correlate to visible color shifts. In general, when applying non-linear methods the transformation matrix needs to be recalculated each time exposure is altered, although recently, some solutions have been proposed for retaining scalability, while increasing the number of free parameters [6,25,26].

Similarly, the planes of constant hue in an *RGB* image, *i.e.*, the plane of *RGB* responses to a matte surface with some elements in light and some in shadow, will be preserved by a linear transformation, but with a non-linear transform, there may be induced color shifts. This issue is covered in detail in [6], where a method is proposed to circumvent this problem.

We note here that the scalability and constant hue plane properties follow directly from the linear response to light of CCD/CMOS sensors [27]. Any non-linearity is added by in-camera image processing, such as gamma correction or noise suppression. It is therefore more practical to directly capture the images in RAW mode, thus circumventing these operations.

## 3. Spherical Sampling for Camera Characterization

The goal of the present paper is to build a 3 × 3 matrix transform that can minimize perceptual errors, rather than errors in *XY Z* space. Human color perception is a non-linear phenomenon, and therefore, methods based on the minimization of a linear measure, such as least-squares with the *L*_2_ norm, are not well adapted to this goal. To achieve this, we need a method for performing a constrained search of the space of possible sensors.

Spherical sampling [11,28,29] provides a means for discretely sampling points on a sphere and relating them to sensors. In particular, given a set of sensors, spherical sampling converts each sensor into a point in the sphere and, from there, samples discrete points on the sphere within a pre-specified angular distance from the starting point. Each of these sample points represents a new sensor. Therefore, for example, given a set of three *RGB* sensors, these can be mapped to three separate points on a sphere. Multiple points, say 10,000, can then be sampled close to each sensor on the sphere. In this example, this results in 10,000 new R sensors, 10,000 new G sensors and 10,000 new B sensors. A discrete set of plausible sensors can then be generated by enumerating all possible combinations of the R, G and B sensors. These potential sensors can then be compared using an error metric.

There are alternative methods to exploring the sensor space. For example, one could sample the nine coefficients of the transform matrix *T* directly or use a gradient descent or direct search algorithm to optimize matrix coefficients. However, spherical sampling has several practical and theoretical advantages.

First, the problem of uniform sampling on a sphere is well understood: we can be sure that sampled points are equally distributed in all different directions, *i.e.*, without over- or under-sampling in any given direction. Sampling the matrix *T* does not ensure even sampling. By adding more data points to mitigate under-sampling (and to ensure a maximum difference between samples), convergence times would be increased and other regions would be unnecessarily over-sampled. Similarly, reducing the number of sample points to avoid over-sampling may miss local error minima. Furthermore, it was shown in [11] that the direct search and gradient descent methods for optimizing coefficients are more likely to fall into sub-optimal error minima than the spherical sampling approach.

Secondly, using spherical sampling, it is possible to define the extent over which the sampling will take place in the sensor space, since the number of points that are sampled in the sphere is directly related to the mean distance between any two solutions. Once again, this information is not available by directly sampling on the matrix, since the same increment in different coefficients of the matrix might represent very different distances in terms of the resulting sensors.

Finally, spherical sampling allows us to define the distance from the starting point with a simple and intuitive measure: the angular distance. Using this distance, it is possible to avoid solutions that are too far away from the starting point. This has been shown to be important in other applications, such as color ratio stabilizations [11], where it was shown that failing to enforce a distance constraint could result in extreme sensor shifts, such as a green sensor being modified to a red one.

### Details of the Method

The problem of camera characterization can be thought of as the conversion from an original set of *RGB* sensors to a different set, *XYZ*. In particular, in the domain of camera characterization, the problem is to map each camera response *RGB* to an *XYZ* tristimulus value. When using a 3 × 3 linear transform, each new coordinate is a linear combination of *RGB*, with weights given by a column of the transform matrix, e.g.,

(3) $$X=t_{11}R+t_{21}G+t_{31}B$$

with the appropriate processing (which we discuss in the next paragraph), the vector [*t*_11_*t*_21_*t*_31_] (along with the other columns of the transform *T*) can be considered as a point on a sphere. Spherical sampling then provides a method for systematically exploring sensor sets by choosing three points on the sphere as the three columns of the transform matrix.

Mathematically, let us represent our original camera sensors *S* as an *m* × 3 matrix, where *m* denotes the number of discrete wavelengths at which the sensor functions are sampled (this value is typically 31, which comes from sampling the visible spectrum 400 nm to 700 nm at 10-nm intervals) and three is the number of sensors, which for most applications, corresponds to the red, green and blue color channels. We perform the reduced singular value decomposition (SVD) of these sensors in order to obtain an orthonormal sensor basis. An orthonormal basis is needed, since differences between coefficient vectors in *T* should map to the same differences in the sensors.

(4) $$S=U\cdot \Sigma \cdot V^{t}.$$

In this equation *U* is an orthonormal matrix with dimension *m* × 3, Σ is a diagonal 3 × 3 matrix containing the singular values of matrix *S*, *V* is an orthogonal 3×3 matrix and * is the transpose operator. Then, *U* is the basis we seek.

From this basis *U*, we can define a new set of sensors *S̃* (*m* × 3), different from the original sensors *S*, by multiplying the basis by any linear transformation *P* (3 × 3), where *P* consists of three column vectors, $\underline{p_{1}}$, $\underline{p_{2}}$, $\underline{p_{3}}$, that are sampled over the two-sphere. Then,

(5) $$\begin{array}{cc} \tilde{S}=UP, & P=\left[\underline{p_{1}},\underline{p_{2}},\underline{p_{3}}\right]; \end{array}$$

we are interested in the relation between the original sensors *S* and the newly defined sensors *S̃*. Using Equations (4) and (5), we have:

(6) $$\tilde{S}=UP=W\Sigma V^{t}{\left(\Sigma V^{t}\right)}^{-1}P=S{\left(\Sigma V^{t}\right)}^{-1}P.$$

Now, replacing this into Equation (2), we obtain:

(7) $$T={\left(\Sigma V^{t}\right)}^{-1}P.$$

We can also rearrange this equation in order to relate a transformation matrix *T* with a set of points *P* over the sphere.

(8) $$P=\Sigma V^{t}T.$$

**Figure 2.**
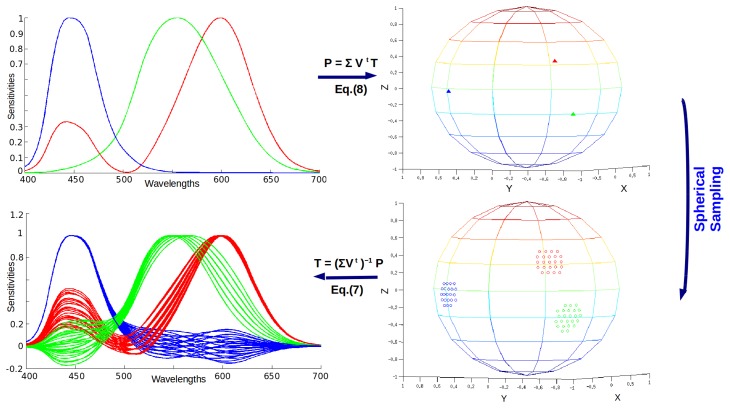
Pipeline of the spherical sampling method. Adapted from [11]. From the original set of sensors, representatives in the sphere are obtained. Then, a sampling over those points in the sphere is performed. Finally, all of the points added are transformed back to the sensor domain, representing a new set of sensors.

A visual explanation of this procedure can be seen in Figure 2.

To find a global error minimum, it is possible, in principle, to sample sensors across the whole surface of the sphere. However, the combination of three separate sensors means that the number of possible three-sensor combinations for *N* samples would be *O*(*N*^3^), which would be prohibitive to compute for large *N*. For example, if we select *N* to be 25, 000, which represents an angular distance between points in the sphere of 1°, we will have 25, 000^3^ = 2.6 trillion sensor sets. Furthermore, it is clear that some sensor choices would be clearly unsuitable, both in terms of generating high error and in some degenerate cases, where the transform matrix becomes rank-reduced and maps *RGB* values to a plane or vector, rather than a complete 3D color space.

For these reasons, instead of sampling all of the sphere, we look at the two last equations for a simplified solution. We start by considering *T_LS_*, which is the solution to Equation (2), which minimizes the least-squares characterization error in *XYZ* space, which is computed by solving the normal equation. Once this matrix is found, we apply Equation (8) to obtain the *T_LS_* representatives in the sphere, and we sample in their surrounds. Details of the sampling scheme, including the number of points and the size of the sampling neighborhood, are provided in the following section. The sampling on the sphere is based on [30].

## 4. Experiments and Results

We have performed three different experiments to demonstrate the capability of our method, each using a different error metric. The first experiment deals with a pixel-wise measure, the CIE Δ*E*. This is, to the best of our knowledge, the only perceptual error metric that has been used, until now, to optimize camera characterization [31]. The other two experiments that we perform, which are based on minimizing a color appearance model (S-CIELAB) and an image quality metric (CID), are novel. They characterize the camera not only in terms of isolated pixel values, but also considering the context (*i.e.*, the spatial arrangement of colors in the scene); a strategy that is more consistent with human vision and which also demonstrates the power of the method to take into account more complex color appearance phenomena.

Our experiments have been performed using simulated camera systems, whereby the camera sensitivities have been measured and recorded in advance, and the illumination and reflectance spectra of the scene are taken from existing databases of spectra and multispectral images.

The simulation works as follows: for each image (or set of pixels), we know the reflectance spectrum at each pixel, and from this, we compute both the *XYZ* value at each pixel and the *RGB* response of the camera using the standard image formation equations. Then, the *RGB* camera responses are converted to estimated *XYZ* by the different methods. Therefore, we have the real and the estimated *XYZ* images, and we calculate the different metrics from these.

To evaluate the device dependency of our results, we use a total of 37 cameras, including 28 used by Jiang *et al.* [32] and nine from the image engineering web page [33]. While we use these cameras to simulate the capture of still images, the characterization process applies equally to video images. For all three experiments, we have computed the matrix *T_LS_*, which minimizes Equation (2) in the least-squares sense, using 102 illuminants and 1995 reflectances from the Simon Fraser University dataset [34]. The spherical sampling procedure considers points within a distance of 3.3° from those representing the starting sensors in the first experiment, and 2.5° for the second and third; we sample 30, 000 points in the sphere in all of the cases, except in Experiment 1b, where we sample 50, 000 points. Let us note again here that with a bigger angular distance and a larger spherical sampling resolution, better results might still be obtained at the cost of extra computations. Our selection of distances and points followed a trade-off between computational time and improvement of the method. However, slight modification of these parameters will not drastically modify our results.

### 4.1. Experiment 1a: CIE ΔE

As we mentioned earlier, *XYZ* is not a perceptual space, *i.e.*, the same distance between two points in different regions of the space represent different perceived distances by a human observer. As a result, minimizing error in *XYZ* space does not necessarily correspond to a minimized perceptual error. In order to avoid this issue, different perceptual color spaces have been defined, most notably: CIELAB, CIELUV [35] and CIECAM [19]. The most commonly-used space is the CIELAB space, as in this space, Euclidean distance correlates with perceptual distance, and the color conversion from *XYZ* to CIELAB is straightforward. The Euclidean distance in CIELAB space is referred to as the CIE Δ*E* and is defined mathematically as:

(9) $$\Delta E\left(\underline{p_{1}},\underline{p_{2}}\right)={\left\|\underline{p_{1}}-\underline{p_{2}}\right\|}_{2}$$

where *p̱*_1_ and *p̱*_2_ are two points in the CIELAB space with dimensions 3×1.

More recently, some improvements on this metric have been proposed that are better correlated with perceptual differences, notably CIE Δ*E* 2000 [20], which is known to give more accurate results for blue colors. The formula for this calculation can be found in [20], and we denote the result as Δ*E*_00_ [36].

In this section, our goal is to minimize, for each particular camera, the average perceptual error for a set of illuminants and reflectances, and we report results for both the Δ*E* and the Δ*E*_00_ metrics.

Mathematically, for a particular camera, we call *p̱_i,j_* (of dimension 3×1) the real *XYZ* value of the pixel for the reflectance *i* ∈ *I* under illuminant *j* ∈ *J* and *q̱_i,j_* (3×1) the *RGB* camera value of the pixel. We look for the matrix *T* that minimizes:

(10) $$\underset{T}{\arg\min}\frac{\sum_{i\in I}\sum_{j\in J}\Delta E(\text{Lab}\left({\underline{p}}_{i,j}^{t}\right),Lab\left({\underline{q}}_{i,j}^{t}\cdot T\right)}{\#I\cdot \#J}$$

where *Lab*() represents the transformation from *XYZ* to CIELAB. We follow the same procedure to calculate Δ*E*_00_, by replacing Equation (9) with the standard formula in [20].

We note here that a similar experiment regarding color constancy using sharp sensors was performed in [11].

We have performed our experiment using the 1995 reflectances and 102 illuminants described above. Computing all possible surfaces under all lights induces a total of 203,490 color signals. We separate these into training and test sets by assigning 90% of the color signals at random to the training set, and the remaining 10% to the test set. We repeat this procedure 100 times to avoid any bias caused by the random selection. In all figures and tables presented in this paper, the error statistics are reported for the test set data only.

**Figure 3.**
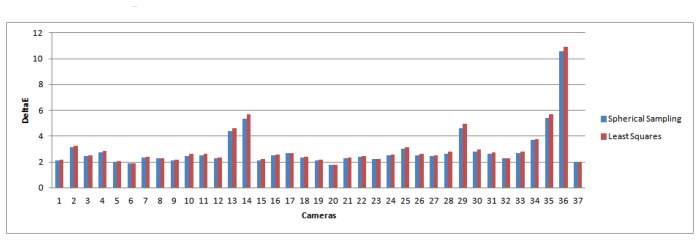
Δ*E* error for the different cameras. In blue: Δ*E* for spherical sampling; in red: error for the least-squares minimization.

**Figure 4.**
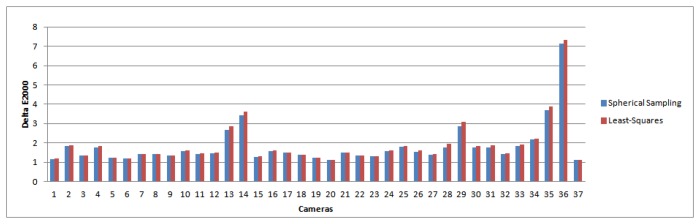
Δ*E*_00_ error for the different cameras. In blue: Δ*E*_00_ for spherical sampling; in red: error for the least-squares minimization.

The results for all 37 cameras are reported in Figures 3 and 4 for the Δ*E* and ΔE_00_ metrics, respectively. Tables 1 and 2 show error statistics relating to the Figures, presenting the improvement obtained in the minimum, maximum, mean and median statistics. In the Tables, the errors for both the least-squares method and our spherical sampling approach are averaged over all of the cameras.

#### 4.1.1. On the Significance of the Results

From these Figures and Tables, it is clear that the spherical sampling approach offers an improvement on the performance of the least-squares method. The main reason for this is that spherical sampling can map *RGB* to *XY Z*, while minimizing error in CIELAB co-ordinates, whereas the least-squares method is minimizing error in *XY Z* space only. While modifications of the least-squares method are plausible using non-linear optimization, this modification is non-trivial, and the spherical sampling method allows us to perform the same minimization directly.

**Figure 5.**
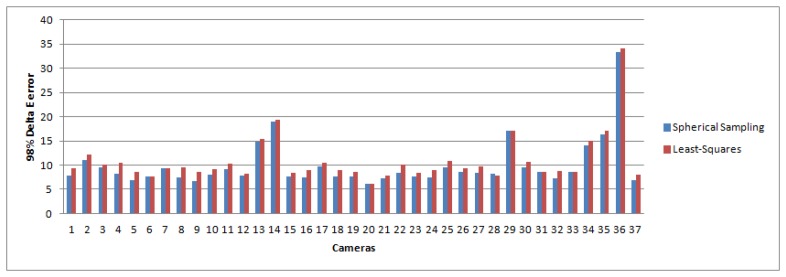
Ninety eighth percentiles per camera for both spherical sampling (blue) and least-squares (red).

**Figure 6.**
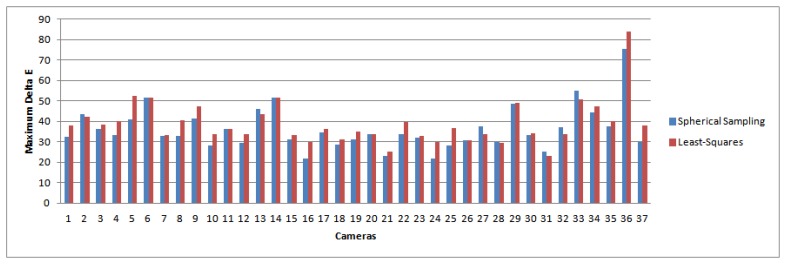
Maxima per camera for both spherical sampling (blue) and least-squares (red).

We note that the magnitude in the improvement over least-squares is consistent with other works presented in the color research literature [37,38]. Furthermore, other statistics suggest that the improvement will have a visual impact. In Figures 5 and 6, we plot the maximum and the 98th percentile of error scores for each camera, which show that the spherical sampling outperforms the least-squares approach by a noticeable margin in a vast majority of the cases.

**Figure 7.**
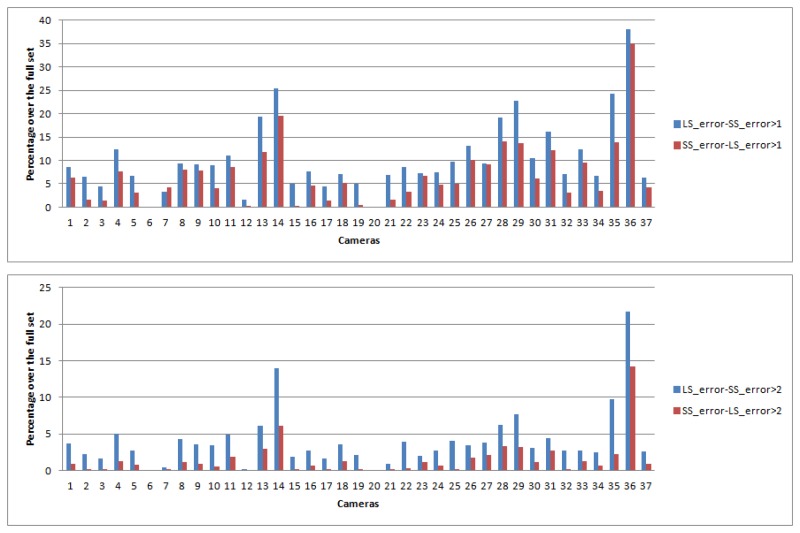
(**Top**) Blue bars: percentage of color signals where spherical sampling gives a lower error than least-squares by 1 Δ*E*. Red bars: percentage of cameras where least-squares gives a lower error than spherical sampling by 1 Δ*E*; (**Bottom**) Blue bars: percentage of color signals where spherical sampling gives lower error than least-squares by 2 Δ*E*. Red bars: percentage of cameras where least-squares gives lower error than spherical sampling by 2 Δ*E*. The results show that our method obtains a perceptible improvement for around 3% more samples than least-squares for both cases.

Similarly, in Figure 7 we plot the percentage of color signals (*i.e.*, combinations of surfaces and illuminants) where either method (Least-Squares or Spherical Sampling) outperforms the other by at least 1 or 2 Δ*E* units, respectively. We can see how for both cases, spectral sharpening is perceptibly better for around 3% more color signals than the least-squares approach. These results suggest a clear benefit in using our method over least-squares.

#### 4.1.2. Comparison *vs.* Matrix Sampling

As explained in Section 3, spherical sampling has some theoretical advantages over simple matrix sampling. Here, we also compare the two methods numerically in terms of the error they produce. Spherical sampling in the previous experiment generates a mean of 16, 000 possible matrices, with an approximate difference in their values of 0.10 per matrix element. To create an analogous situation for direct matrix sampling, we have randomly obtained 16,000 matrices by directly adjusting the initial least-squares estimate by an increment ranging from −0.05 to 0.05 for each matrix coefficient. In Table 3, we present the average error for the full set of cameras, illuminants and reflectances used. We can see that our method, with a more efficient approach to sampling, gives lower error.

### 4.2. Experiment 1b: Different Illuminants

As we pointed out in the Introduction, some cameras calculate pre-set color correction matrices under different illuminations, which gives better color correction for each individual illuminant. In this section, we look at how well our method works in this situation by deriving separate color correction matrices under different illuminants and comparing the performance of our technique with the least-squares approach, where the least-squares solution is derived using the same illuminant.

**Figure 8.**
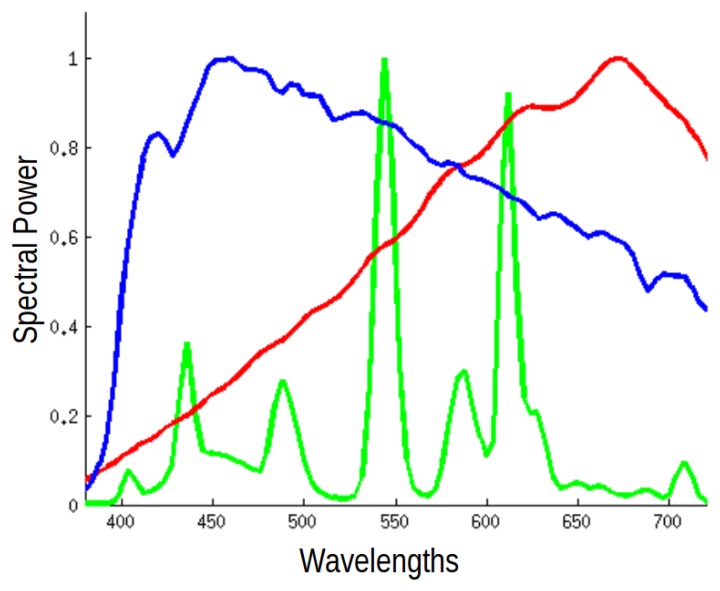
Spectral sensitivities of the three illuminants used in Experiment 1b.

Using the same set of 1995 reflectances, we generated color signals for each of three illuminants: one representing daylight, one a fluorescent illuminant and the third an incandescent illuminant. The spectral sensitivities of these illuminants can be found in Figure 8. For each illuminant, we separate the data into training and test sets, with a proportion of 90% for training and 10% for the test. We then use the spherical sampling procedure to generate a color correction matrix for the training set and to test the performance on the test set and then repeat this process 100 times. Results of this procedure, measured using Δ*E* and Δ*E*_00_ error, are shown in Tables 4 and 5.

### 4.3. Experiment 2: S-CIELAB

Our previous experiments, which can be considered the *de facto* experiments for perceptual minimization, have one main drawback: they do not take into account the image context. For example, when looking at a yellow pixel, the distance between the real and approximated value will be the same, both if it has a pink neighborhood or an orange one. This is known to be false, since human perception relies deeply on the context of the scene [39]. It is for this reason that S-CIELAB was proposed [21]. Basically, S-CIELAB computes the Δ*E* error measure after applying spatial pre-processing to account for the spatial-color sensitivity of human vision. We note here that in practice, our method can be applied to minimizing characterization error for a range of more complex perceptual measures, such as CIECAM02. Here, we use S-CIELAB and another spatial error metric, CID (see below), as test cases to demonstrate the power of the approach, while we acknowledge that resulting spatial biases introduced into the characterization matrix may only be optimal for restricted imaging situations.

**Figure 9.**
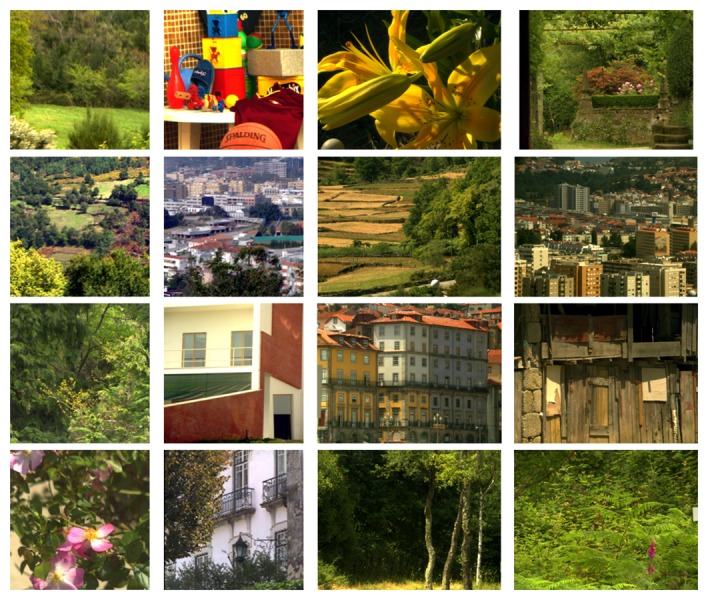
Foster *et al.* [40] hyperspectral dataset used in Experiments 2 and 3.

**Figure 10.**
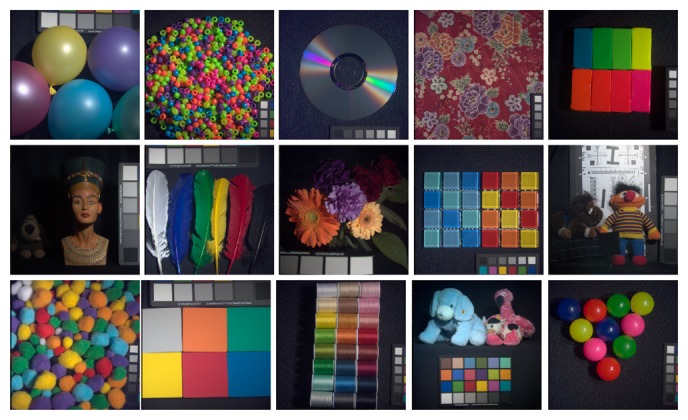
Yasuma *et al.* [41] rendering of the hyperspectral dataset used in Experiments 2 and 3.

Given that S-CIELAB is applied to images, for the purposes of our experiments, it is important to use meaningful images and meaningful illumination. To this end, we have used three different datasets: the 16 hyperspectral images obtained by Foster *et al.* [40], which contain a range of man-made objects, natural landscapes and both indoor and outdoor scenes, the 15 images labeled as “stuff” obtained by Yasuma *et al.* [41], which contain a range of very colorful objects, and the combination of both datasets. We have calculated color signals using the *D*65 illuminant. The 16 images of the first dataset are presented in Figure 9, while the 15 images of the second are presented in Figure 10.

In this experiment, for each camera, we use spherical sampling to calculate a color correction matrix that minimizes S-CIELAB error. Mathematically, if we define *I_h_*_,_*_D_*_65_ (dimension *N* × 3) as the real *XY Z* values of image *h* of the database under the D65 illuminant and *J_h_*_,_*_D_*_65_ (dimension *N* × 3) as the image obtained by the camera for the same image and illuminant, we search for the matrix *T* that minimizes:

(11) $$\arg\underset{T}{\min}\frac{\sum_{h=1}^{M}S‐\text{CIELAB}\left(I_{h,D65},J_{h,D65}\cdot T\right)}{M}$$

To separate the training and test sets we use a leave-one-out procedure, whereby, for each dataset, we use all except one image to build the transform matrix and test the method on the remaining image. We repeat this procedure as many times as there are images in the dataset, leaving out each image in turn and then calculating the mean error over all images.

**Figure 11.**
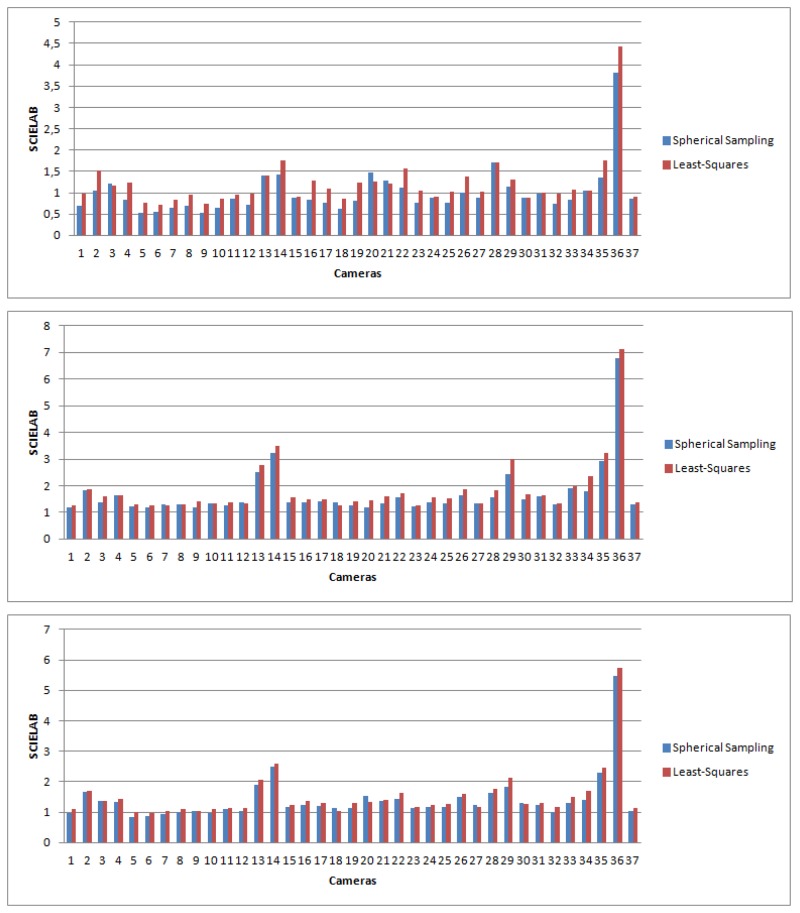
S-CIELAB error for the different cameras computed as the mean for the different images. In blue: S-CIELAB error for spherical sampling; in red: error for the least-squares minimization. (**Top**) First dataset; (**middle**): second dataset; (**bottom**) third dataset.

Results for all of the different cameras on the three different datasets are shown in Figure 11. A statistical analysis of the results is shown in Table 6.

### 4.4. Experiment 3: CID Measure

Lissner et al., have recently proposed a new perceptually based Color Image Difference (CID) metric, which shows a good correlation with human evaluations of gamut mapping algorithms [23]. The method is originally based on the intensity SSIM image quality metric of Wang et al. [42].

In this experiment, we have followed the same procedure as Section 4.3 and have used the leave-one-out paradigm to minimize the mean CID error for a subset of the hyperspectral images and to test a novel image for which the method was not trained.

Defining *I_h,D_*_65_ as the real *XY Z* image *h* of the database under the D65 illuminant and *J_h,D_*_65_ as the image obtained by the camera for the same image and illuminant, we search for the matrix T minimizing:

(12) $$\arg\underset{T}{\min}\frac{\sum_{h=1}^{M}\text{CID}\left(I_{h,D65},J_{h,D65}\cdot T\right)}{M}.$$

**Figure 12.**
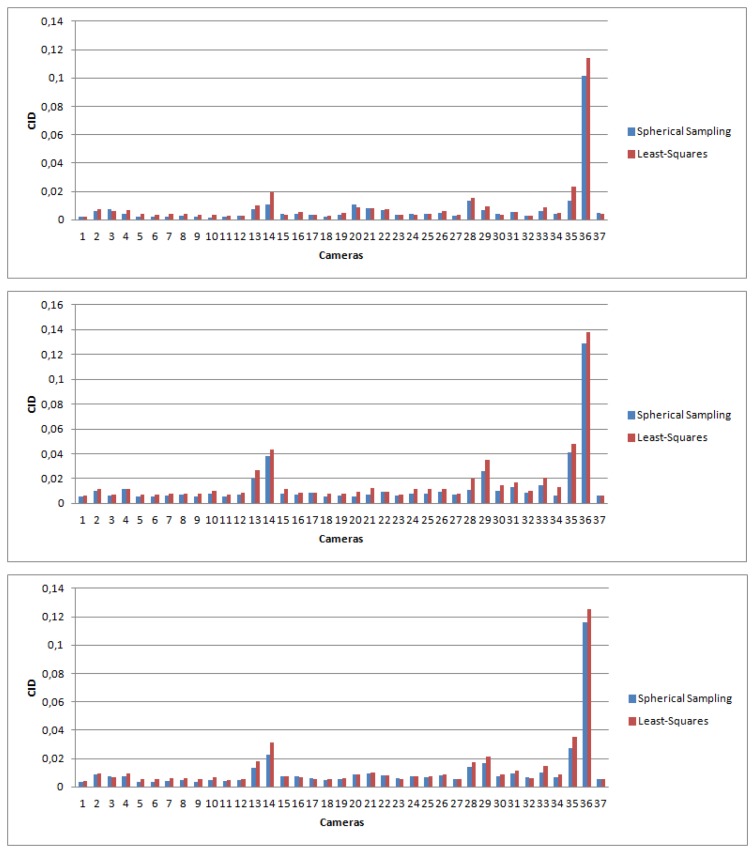
Color Image Difference (CID) error for the different cameras computed as the mean for the different images. In blue: CID error for spherical sampling; in red: error for the least-squares minimization. (**Top**) First dataset; (**middle**) second dataset; (**bottom**): third dataset.

Results are shown in Figure 12, and once again, spherical sampling outperforms the least-squares optimization. A statistical analysis is shown in Table 7.

### 4.5. Qualitative Examples

The previous sections outline results for quantitative metrics that have proven correlations with human perceptual performance in image evaluation. While a full perceptual experiment is beyond the scope of the present work, in this section, we include some visual examples to help understand the method's performance in a more intuitive way.

**Figure 13.**
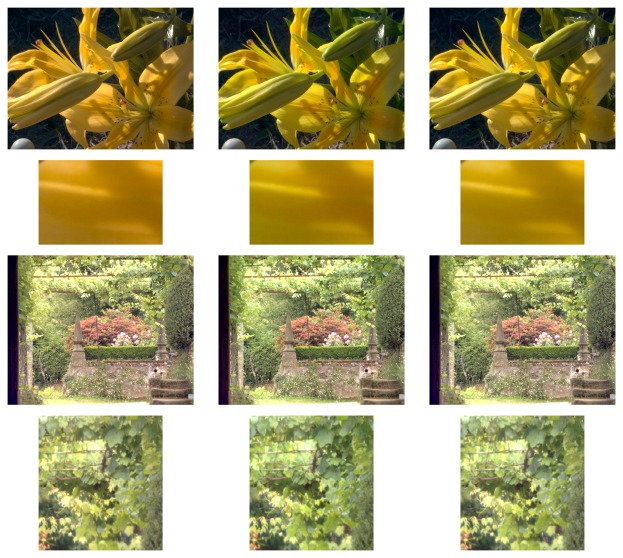
Qualitative evaluation of the approach. Top two rows, minimizing the S-CIELAB measure: full image (**top column**) and detail (**second row**). Last two rows, minimizing the CID measure: full image (**third row**) and detail (**last row**). For each column the images are: original (**left**), least-squares (**center**) and spherical sampling (**right**).

Figure 13 presents some qualitative examples of color correction matrices that minimize both the S-CIELAB and the CID measures. The images are presented in *sRGB* using the standard transform to convert from *XY Z* to *sRGB*. In the top rows, *i.e.*, the flower image, we present color correction results that minimize S-CIELAB error. The image on the left, which is the least-squares minimization, has a mean S-CIELAB difference from the middle image (the real image) of 8.74. The image created by spherical sampling, shown on the right, gives a mean difference of 4.86. The bottom rows, *i.e.*, the garden image, show the color correction results from minimizing the CID measure. The image on the left (the least-squares solution) has a mean difference from the central image (the original) of 0.0569, while the image generated from spherical sampling, shown on the right, gives an error of 0.0232. A cropped version of this comparison is presented in the last row. We note here the yellowish cast in the least-squares image.

**Figure 14.**
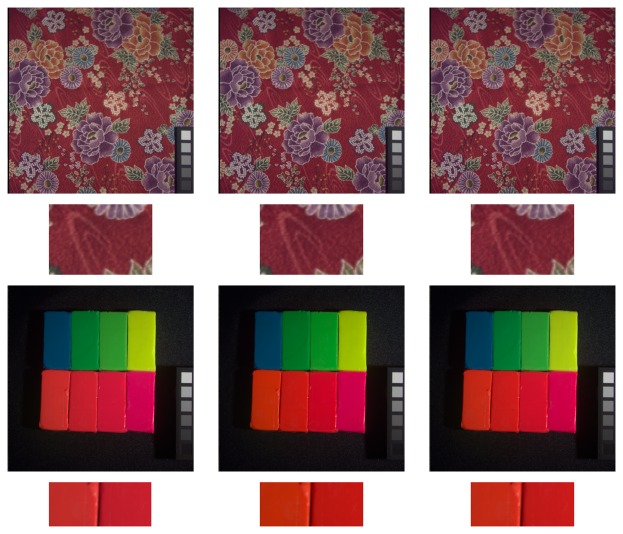
Qualitative evaluation of the approach *versus* matrix sampling. Top two rows, minimizing the S-CIELAB measure: full image (**first row**) and detail (**second row**). Last two rows, minimizing the CID measure: full image (**third row**) and detail (**last row**). For each column, the images are: matrix sampling (**left**), original (**center**) and spherical sampling (**right**).

We also present a qualitative example for comparison between the spherical sampling and matrix sampling approaches in Figure 14. In this figure, the spherical sampling images (right), appear closer to the original images (center) than those from matrix sampling (left), especially in the red colors. In particular, for the S-CIELAB case, matrix sampling gives a mean error for the whole image of 1.2823, while spherical the sampling error is 0.7624. In the case of the CID measure, the mean error using matrix sampling is 0.0248, while the spherical sampling error is significantly lower: 0.0076.

## 5. Discussion

In the previous section, we have minimized three different error metrics independently. In Table 8, we compare the results between these metrics using the mean percentage improvement for the three different cases. As expected, our method shows an improvement when minimizing Δ*E* and Δ*E*_00_, but less of an improvement than it shows for the image-based metrics. Even in this case, our improvement is approximately 3%. More impressive are the results for the other two metrics, where our spherical sampling procedure reduces the error by more than 6% for S-CIELAB and 13% for the CID.

Another way to represent the results is to analyze what is happening in the sensor space. As our method maps XY Z to RGB responses, one can also conceptualize this is as mapping the RGB sensor sensitivities directly to the *XYZ* color matching functions. The question then is: are the approximate *XYZ* color matching functions found by spherical sampling closer to the real *XYZ* sensors than those obtained via the least-squares optimization? To address this question, we have defined a new measure.

Let us define *X*, *Y* and Z as *m* × 1 vectors, which are discrete representations of the color matching functions *x̄*, *ȳ* and *z̄*, respectively, sampled at *m* wavelengths spanning the domain ω*.* Let us also define *X̂^LS^*, *Ŷ^LS^* and *Ẑ^LS^* as the approximation derived by least-squares (LS) and *X̂*^SS^, *Ŷ*^SS^ and *Ẑ*^SS^ as the approximation derived by spherical sampling (SS). We also define $e_{i}^{LS}$ and $e_{i}^{SS}$, *i* ∈ {*X,Y,Z*} as the error obtained for both methods, which we compute for each sensor separately; e.g., the error for the *X* sensor is calculated as:

(13) $$e_{X}^{LS}=\sum_{m\in \omega }{\left\|X_{m}-{\hat{X}}_{m}^{LS}\right\|}_{q}$$

where *q* represents the chosen norm. In this paper, we will use *q* = 1 and *q* = 2. We choose these values, since *q* = 1 represents the response to a flat white surface under a white illuminant and *q* = 2 is the typical Euclidean norm. From the previous equation, the difference between the two approaches can be computed as:

(14) $${\text{diff}}_{X}\left(LS,SS\right)=e_{X}^{LS}-e_{X}^{LS};$$

therefore, when this difference is smaller than zero, the least-squares sensors are closer to the real sensors than spherical sampling ones and *vice versa*.

We note here that while Equation (13) could be minimized more directly by computing a matrix *T* from Equation (1), the purpose here is to compare the transforms derived using real spectra and illuminants for their ability to approximate the *XYZ* color matching functions. This metric provides an indirect measure of how well the methods will generalize to unseen data, and we use it as a “sanity check” to further ensure that overtraining is not occurring.

In Table 9, we show the results averaged over all cameras for all three sensors in the three different experiments. In this case, we have performed the minimization of the three measures on the full set of images, *i.e.*, we do not separate training and test data, since the metric we are computing is different from the metric used to learn the transforms. We can see that the spherical sampling technique results in lower error than the least-squares method in 15 out of 18 cases, which reinforces the adequacy of our method.

## 6. Conclusions

The present work investigates a novel technique for mapping camera *RGB* responses to device independent, CIE *XY Z*, color co-ordinates. The method works by discretely sampling sensor sets that are close to an initial “best-guess” solution derived by minimizing the least-squares error. The discrete nature of the algorithm means that error can be minimized in any chosen color space, which allows the optimization to take into account the spatial characteristics of colors in images and takes a step towards characterizing perceptual, as well as physical aspects of the color.

The method was tested on a range of different images and simulated camera response curves. The results show that for over 90% of image and sensor combinations, the initial least-squares solution can be improved upon by spherical sampling. This improvement ranges from approximately 3% for CIELAB Δ E error to 7% for the S-CIELAB and 13% for the CID error metrics.

This method would be of direct and immediate applicability for camera manufacturers, who would be able to appreciably increase the accuracy of the color characterization of their cameras, without any need to change the experimental set-up that they use. Furthermore, the proposed method is of interest for color researchers and professional photographers/cinematographers, who may use an affordable calibration device to estimate the *RGB* response curves of the camera and then apply our method to properly correct the RAW data.

## Figures and Tables

**Table 1.** Δ*E* results: average for 37 cameras.

	**Least-Squares**	**Spherical Sampling**
Min	**1.7764**	**1.7764**
Max	10.9365	**10.5820**
Mean	3.0289	**2.9270**
Median	2.5801	**2.4692**

**Table 2.** ΔE_00_ results: average for 37 cameras.

	**Least-Squares**	**Spherical Sampling**
Min	**1.1178**	**1.1178**
Max	7.3300	**7.1277**
Mean	1.8784	**1.8245**
Median	1.5049	**1.4950**

**Table 3.** Comparison between matrix sampling and spherical sampling as the mean for all the cameras, illuminants and reflectances.

	**Matrix Sampling**	**Spherical Sampling**
ΔE	2.9555	**2.9270**
ΔE_00_	1.8316	**1.8245**

**Table 4.** Δ*E* error for three different illuminants on the Simon Fraser dataset (Least-Squares (LS), Spherical Sampling (SS)).

	**Daylight**		**Incandescent**		**Fluorescent**	

	**LS**	**SS**	**LS**	**SS**	**LS**	**SS**
Min	**1.0581**	**1.0581**	**0.9804**	**0.9804**	**1.0092**	**1.0092**
Max	6.9781	**6.8166**	6.3126	**6.1703**	4.5314	**4.3278**
Mean	1.8353	**1.8153**	1.6491	**1.6297**	1.7122	**1.6690**

**Table 5.** Δ*E*_00_ error for three different illuminants on the Simon Fraser dataset (Least-Squares (LS), Spherical Sampling (SS)).

	**Daylight**		**Incandescent**		**Fluorescent**	

	**LS**	**SS**	**LS**	**SS**	**LS**	**SS**
Min	**0.6729**	**0.6729**	**0.5527**	**0.5527**	**0.4915**	**0.4915**
Max	4.5663	**4.4134**	4.3101	**4.2190**	2.5441	**2.4224**
Mean	1.1240	**1.1116**	0.9465	**0.9330**	0.8057	**0.7967**

**Table 6.** S-CIELAB results: average result for 37 cameras using a leave-one-out paradigm (Least-Squares (LS), Spherical Sampling (SS)).

	**Foster *et al.***		**Yasuma *et al.***		**Combination**	

	**LS**	**SS**	**LS**	**SS**	**LS**	**SS**
Min	0.7139	**0.5173**	1.2528	**1.1716**	0.9766	**0.8395**
Max	4.4245	**3.8121**	7.1496	**6.7660**	5.7788	**5.4829**
Mean	1.2100	**1.0056**	1.8447	**1.6981**	1.5170	**1.4154**
Median	1.0529	**0.8701**	1.5141	**1.3740**	1.2856	**1.2240**

**Table 7.** CID results: average for 37 cameras.

	**Foster *et al.***		**Yasuma *et al.***		**Combination**	

	**LS**	**SS**	**LS**	**SS**	**LS**	**SS**
Min	0.0024	**0.0018**	0.0061	**0.0053**	0.0041	**0.0036**
Max	0.1144	**0.1017**	0.1377	**0.1288**	0.1256	**0.1157**
Mean	0.0092	**0.0076**	0.0166	**0.0136**	0.0127	**0.0106**
Median	0.0045	**0.0040**	0.0098	**0.0077**	0.0074	**0.0072**

**Table 8.** Percentage improvement in error-metric for spherical sampling when compared to least-squares.

	**Δ*E***	**Δ*E*_00_**	**S-CIELAB**	**CID**
Mean percentage	3.42%	2.87%	6.90%	13.50%

**Table 9.** Error in a least-squares optimization minus error using a spherical sampling procedure computed in the sensor space.

	**Δ*E***			**S-CIELAB**			**CID**		

	**X**	**Y**	**Z**	**X**	**Y**	**Z**	**X**	**Y**	**Z**
L1	1.03	0.20	0.33	0.31	0.08	0.94	0.40	0.15	0.55
L2	1.43	0.30	–0.04	0.36	0.01	–0.38	0.47	0.11	–0.39
